# Benchmarking Highly Parallel Hardware for Spiking Neural Networks in Robotics

**DOI:** 10.3389/fnins.2021.667011

**Published:** 2021-06-29

**Authors:** Lea Steffen, Robin Koch, Stefan Ulbrich, Sven Nitzsche, Arne Roennau, Rüdiger Dillmann

**Affiliations:** Interactive Diagnosis and Service Systems (IDS), Intelligent Systems and Production Engineering (ISPE), FZI Research Center for Information Technology, Karlsruhe, Germany

**Keywords:** spiking neural networks, parallel hardware architectures, benchmark, robotic motion control, neurorobotic

## Abstract

Animal brains still outperform even the most performant machines with significantly lower speed. Nonetheless, impressive progress has been made in robotics in the areas of vision, motion- and path planning in the last decades. Brain-inspired Spiking Neural Networks (SNN) and the parallel hardware necessary to exploit their full potential have promising features for robotic application. Besides the most obvious platform for deploying SNN, brain-inspired neuromorphic hardware, Graphical Processing Units (GPU) are well capable of parallel computing as well. Libraries for generating CUDA-optimized code, like GeNN and affordable embedded systems make them an attractive alternative due to their low price and availability. While a few performance tests exist, there has been a lack of benchmarks targeting robotic applications. We compare the performance of a neural Wavefront algorithm as a representative of use cases in robotics on different hardware suitable for running SNN simulations. The SNN used for this benchmark is modeled in the simulator-independent declarative language PyNN, which allows using the same model for different simulator backends. Our emphasis is the comparison between Nest, running on serial CPU, SpiNNaker, as a representative of neuromorphic hardware, and an implementation in GeNN. Beyond that, we also investigate the differences of GeNN deployed to different hardware. A comparison between the different simulators and hardware is performed with regard to total simulation time, average energy consumption per run, and the length of the resulting path. We hope that the insights gained about performance details of parallel hardware solutions contribute to developing more efficient SNN implementations for robotics.

## 1. Introduction

Although impressive progress has been made in robotics, in the areas of perception and motor control, animal brains with significantly less performant components still outperform even the most sophisticated machines. While brain inspired research with Spiking Neural Networks (SNNs) and neuromorphic sensors shows great potential, their slow execution on common simulation frameworks running on serial CPUs prevent their broad application in robotic areas as vision, motion-, and path planning up to date.

As performing the updates for every neuron in parallel significantly reduces simulation time, hardware enabling massive parallelism enables the full exploitation of SNNs' capabilities. In order to allow researchers and developers to make a good choice regarding the software and hardware solutions when working with spiking neurons, comprehensive benchmarks are required.

Inspired by the nervous system, highly parallel platforms have been developed targeting low-power, large-scale SNN simulations in real time. The basis for biomimetic or neuromorphic hardware is the observation that the operation principles of information processing in nature differ greatly from artificial methods. In most cases, artificial methods are significantly less effective than their biological counterpart, for which reason scientists for instance, started to investigate retinal computation (Mead, 1990), inspired by processing in the brain. Well known representatives of this technology are IBMs TrueNorth chip (Merolla et al., 2014), Intel's Loihi (Davies et al., 2018), the SpiNNaker system of the University of Manchester (Furber et al., 2014), and BrainScaleS developed in Heidelberg (Friedmann et al., 2016).

TrueNorth is a digital neuromorphic chip that supports the simulation of up to 1 million neurons and 256 million synapses per chip. While the chip has a high density of neurons and synapses, one is limited to use the LIF neuron model and the synapses are static (Merolla et al., 2014). Loihi chips consist of 128 neuromorphic cores capable of simulating up to 1,024 LIF neurons each. The Loihi chip is fully digital and supports dynamic synapses (Davies et al., 2018). SpiNNaker is a fully digital neuromorphic system developed for large-scale simulations of SNNs. As it is composed of general-purpose ARM microprocessors, neuron and synapse models can be specified and adapted by software, making it very flexible (Furber et al., 2014). In Mayr et al. (2019), the second generation SpiNNaker-2, featuring 10 Million instead of 1 Million cores, is introduced. BrainScaleS, which is derived from the single-chip implementation Spikey (Schemmel et al., 2006), is a neuromorphic mixed-signal chip. In contrast to many others, BrainScaleS and its successor, BrainScaleS 2 are analog (Müller et al., 2020a,b).

Besides these popular candidates of non-von Neumann computing a multiplicity of neuromorphic hardware has been developed in the last decade. In Yan et al. (2021), a differentiation in 3 classes is made; (1) systems with static synapses like TrueNorth, NeuroGrid, Braindrop, HiAER-IFAT, DYNAPs, Tianjic, NeuroSoC, and DeepSouth, (2) systems supporting a configurable plasticity like ROLLS, ODIN, and TITAN and lastly, (3) systems supporting a programmable plasticity like both BrainScaleS, both SpiNNaker and Loihi. Another digital representative is focused on memory centric computing is Neurocube (Kim et al., 2016). However, since the development is progressing rapidly, a comprehensive list is difficult and quickly outdated. Comparatively new developments are the large-scale neuromorphic architectures CerebelluMorphic (Yang et al., 2021b) and BiCoSS (Yang et al., 2021a). CerebelluMorphic is a cerebellum-inspired neuromorphic architecture. Since the cerebellum is crucial for motor control, this technology is very interesting for robotics.

Another type of hardware well-suited for parallel computing is the Graphical Processing Unit (GPU), which was originally developed for computer game graphics. The introduction of high level programming languages such as CUDA or OpenCL, allowed GPUs to be used for general purpose parallel programming. This trend is supported by the development of accessible, inexpensive embedded systems like Nvidia's Jetson boards. Already in 2010 a parallel implementation of a SNN on NVidia CUDA showed a significant speed up (Nowotny, 2010). This idea was further investigated and in Yavuz et al. (2016) GPU enhanced neuronal networks (GeNN) a tool for code generation for specifying ANNs, especially focusing on SNNs, is introduced. It provides a simple C++ API generating optimized C++ and CUDA code. GeNN includes as well a C++ backend and CUDA backend and additional python module (PyGeNN) to support TensorFlow and PyNN. Further methods to execute ANNs on GPU are presented in Minkovich et al. (2014) and Mutch (2010). As stated in Vineyard et al. (2019), techniques for comparing neuromorphic architectures and similar systems are vital, as many event-driven methods may be well-suited for some, but inapplicable for others.

In Blundell et al. (2018), methods for code generation in computational neuroscience are reviewed and respective simulators, modeling languages, and frameworks are introduced and assessed. The authors cover, amongst others, code generations for a variety of hardware and software solutions like the neural simulators Brian (Stimberg et al., 2020), NEST (Gewaltig and Diesmann, 2007), NEURON (Carnevale and Hines, 2006), and GENESIS (Bower et al., 1998) running on serial CPU as well as techniques focusing on the execution on NVIDIA GPUs as GeNN (Yavuz et al., 2016) and Myriad (Rittner and Cleland, 2014). Furthermore, code generation for the neuromorphic hardware SpiNNaker (Furber et al., 2014) and the high-performance computing platform The Virtual Brain (TVB-HPC) (Sanzleon et al., 2013) are included.

In 2018, two benchmarks focusing on a neuroscientific use case, a cortical microcircuit model, have been presented. In van Albada et al. (2018), the models are implemented in PyNN and the performance of the SpiNNaker system running 6 SpiNN-5 boards and NEST running on a HPC cluster is compared. While the focus of the benchmark is on the accuracy of the simulation results, the total simulation time and energy per synaptic event are evaluated as well. Configurations of the HPC tuned for low energy consumption and simulation speed perform better than the SpiNNaker system. The simulations in both NEST- and SpiNNaker implementations are similar with regard to accuracy, hence showing the capabilities of the SpiNNaker system to perform large scale simulations. In contrast to van Albada et al. (2018), Knight and Nowotny (2018) uses C++ for simulating the cortical microcircuit model with GeNN on different pieces of hardware and comparing the performance to the SpiNNaker and NEST implementations. The authors state that—at least for their use case—certain GPUs outperform HPC systems as well as neuromorphic hardware regarding energy consumption and speed. As the accuracy of the simulation in GeNN is also comparable to the NEST implementation, GPUs are shown to be suitable architectures for SNN simulations that are able to compete with neuromorphic hardware. The authors also outline the possibility of using GPU-based SNN controllers in robots. Benchmark scenarios more focused on a task applicable outside of the neuroscientific community are carried out in Diamond et al. (2016) and Ostrau et al. (2020). Both apply image classification and evaluate their models by using the MNIST data set. The former is executed on the neuromorphic systems Spikey and SpiNNaker, as well as GeNN. The latter uses Cypress and therefore the PyNN interface for NEST, Spikey and SpiNNaker and for GeNN and BrainScaleS the respective C++ interfaces are applied. In Diamond et al. (2016), the SNN used for image detection is a model of the olfactory system described in Schmuker and Schneider (2007).

As the work of Ostrau et al. (2020) shows some similarities to our work, it is noteworthy that they pre-learn several conventional ANNs which are transformed into SNNs. It is shown that the SpiNNaker system can be efficient if it is used to its full extend, whereas the GeNN implementations are most suitable if one focuses primarily on short simulation times.

Very recently, a comparison between Loihi and a prototype of SpiNNaker 2 has been done in Yan et al. (2021). Two kind of benchmark tasks are used, keyword spotting and adaptive robotic control to compare the hardware regarding power consumption and computation time. The authors conclude that a general statement is not possible as the energy efficiency is highly influenced by the number of input dimensions. As SpiNNaker 2 handles high-dimensional vector-matrices better, it is faster and more energy-efficient for keyword spotting. Loihi is superior in regard to less complicated vector-matrix multiplication. Furthermore, in DeWolf et al. (2020) a development workflow, targeting neurorobotics applications, running on standard as well as neuromorphic hardware, is presented. The authors illustrate how Nengo helps users to develop robotic sensor and actor applications, using two examples. The work creates a basis for benchmarking neuromorphic architecture, specifically Loihi, against standard hardware regarding robotic applications implemented in Nengo.

Even though applying SNNs to robotic use cases is very promising and despite the necessity of dedicated hardware for fast and resource-friendly execution, the performance of parallel hardware for SNNs has not been analyzed in the context of robotics sufficiently. One particular area of robotics, path finding, has not been investigated yet. In Davies (2019), an article giving guidance for benchmarking neuromorphic hardware, the temporal wavefront propagation was rated as an interesting candidate by name, as it is seen as a viable contribution to the greater field of neuromorphic benchmarks. While the authors of Yan et al. (2021) include a robotics scenario, their benchmark is limited to neuromorphic hardware. However, to develop efficient robotics solutions with SNNs, it is crucial to know specification- and performance-related details of all accessible systems to make an informed decision regarding hardware. Hence, we focus on an application-oriented robotic scenario. Using PyNN enables us to include representatives of GPU-based and neuromorphic computing as well as conventional simulators. Furthermore, this performance comparison covers several different GPU-based hardware realizations as the GeNN implementation is run on three candidates of the Jetson series by Nvidia.

In this work, we carry out a benchmark of hardware well suited for SNN simulation with an application-oriented test scenario intended to be used in robotics.

## 2. Methods

To correctly derive how the different systems perform in comparison, it is crucial to run the experiments with a realistic workload. As this work compares parallel benchmarks for robotic applications, we chose the 3D neural path planning (Steffen et al., 2020), described in section 2.1, as the test scenario. It was chosen because path finding and motion control are corner stones for robotics. Due to their strong synergies, hardware and software in brain-inspired systems need to be considered together when selecting suitable candidates for benchmarking. In section 2.2, the decision-making process and its outcome are set out. As the architecture of parallel hardware, especially neuromorphic, is very different from the von Neumann architecture (VA), traditional benchmarks are hardly transferable to event-driven spiking use cases. Hence, meaningful metrics as introduced in section 2.3 are necessary.

### 2.1. A Robotic Scenario—The Wavefront Algorithm

A popular method for pathfinding is the so-called Wavefront algorithm. It represents the environment as a matrix Map(i, j). Each free cell has a value assigned to it which represents its distance from the target cell and is also referred to as the weight. Its value corresponds to the minimal value of its neighboring cells +1. If a cell is occupied it is not assigned value. This can be formalized by:

(1) $$\begin{array}{l} Map(i,j)=\{\begin{array}{ll} min(neighborhood(i,j))+1 & if\;empty\\ nothing & if\;full \end{array} \end{array}$$

In order to find the shortest path the mobile agent then simply follows the cells with the smallest weights until it reaches its target (Nooraliei and Nooraliei, 2009; Pal et al., 2011).

**Figure 1:**
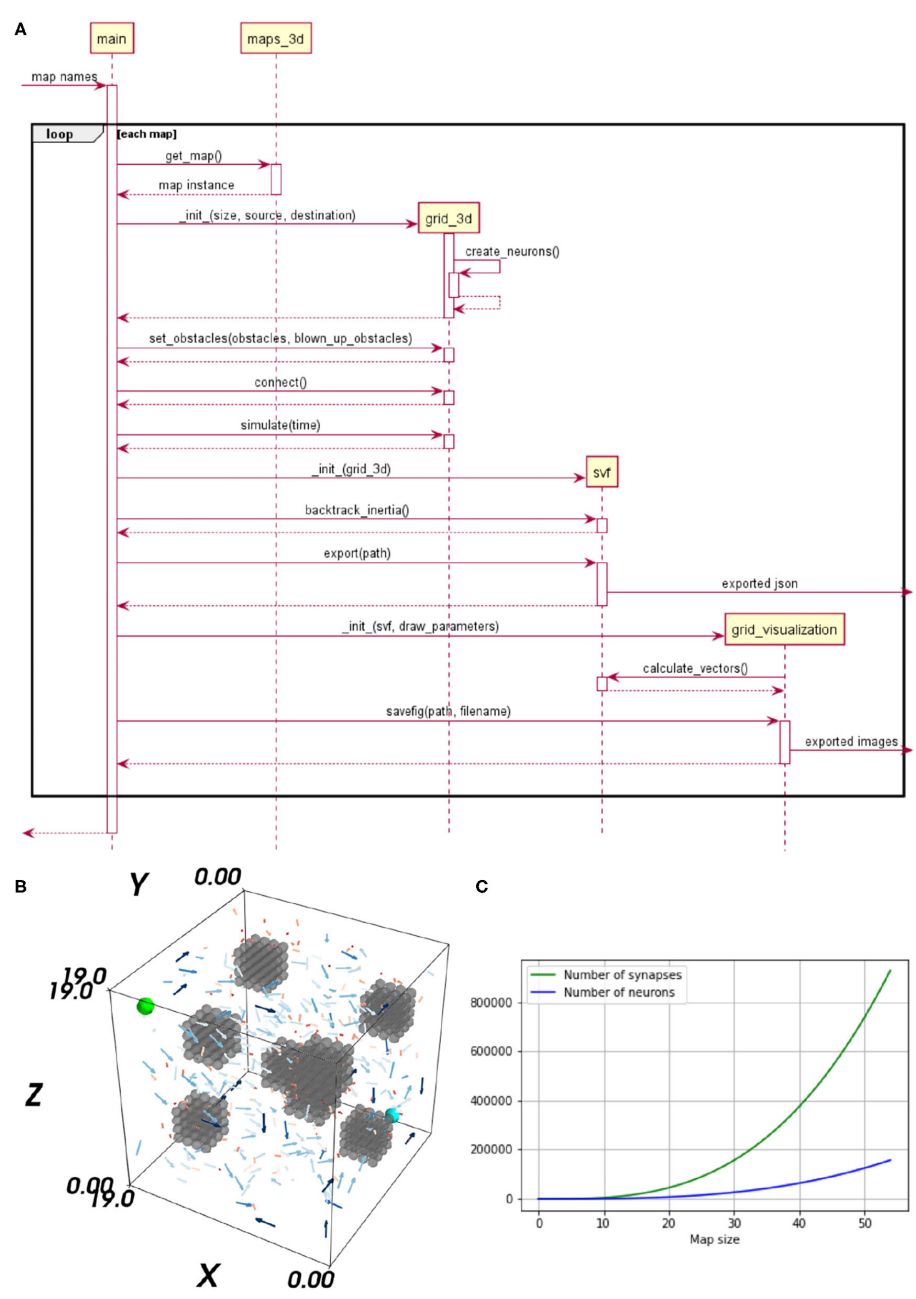
**(A)** Sequence diagram of the neural Wavefront algorithm for robot motion control, as presented in Steffen et al. (2020). **(B)** A vector field's flow on a map including three obstacles. The start neuron is marked in cyan and the target neuron in light green. **(C)** Impact of an up scaled map on the number of neurons and synapses is shown. The x-axis represents the map size and the y-axis shows the number of neurons and synapses, respectively.

In Steffen et al. (2020), the neural path planning algorithm for robotic motion control is introduced. This implementation is the test scenario of our benchmark. The method generates a synaptic vector field (SVF), revealing a path, by propagating a wavefront on a 3D environment. The environment is represented as a cognitive map, a grid of excitatory place cells realized as an SNN. The method, based on the 2D variation proposed in Ponulak and Hopfield (2013), applies bio-inspired techniques and is especially interesting for reactive flexible motion control as needed for Human-robot interaction. The implementation of Steffen et al. (2020) is carried out in NEST and tested on maps with varying degrees of complexity. The NEST implementation already allows fast simulation and query times but shows strong weaknesses regarding the creation time. The purposeful use of dedicated hardware, allowing massive parallelism, shall overcome these issues. A detailed visualization of the implemented algorithm is given as a sequence diagram in Figure 1A. As the evaluation in 3 embodies a specific analysis of the algorithm's sub-tasks, a brief explanation of the significant sub-processes is provided.

#### 2.1.1. A Cognitive Map

The neural environment representation is realized as an SNN using place cells. The network's topology corresponds to a discretization of the environment. Each voxel is translated as a place cell implemented as a single neuron. The network's neurons are connected via the Manhattan method, solely supporting lateral connections. As we use bi-directional connections the synapses are not symmetrical. The neurons representing free and occupied space are identical. However, neurons embodying free space are connected by excitatory synapses and neurons embodying obstacles by inhibitory ones.

#### 2.1.2. Synaptic Vector Field

The SVF is an interpretation of the synapses' weights, of the trained network. For learning spike-timing-dependent plasticity (STDP), a biologically plausible learning rule updating the weights depending on the precise pre-synaptic and post-synaptic spike times (Gütig et al., 2003) is used. To generate a SVF with neural waves, three steps are required. *(1) Initialization*, the place cell representing the target position triggers the neural wave. Applying an electrical current to the respective neuron, increases its membrane potential. Thereby a spike is emitted, exciting the neuron's neighbors and so starting the wave of activation which evolves through the neural grid. *(2) Learning*, through the learning rule STDP the synaptic weights are altered in the direction of the wave. *(3) Interpretation*, by retrieving the synapses' weights as vectors, a vector field is generated. This vector field is used for visualization purposes and for finding a path. In Figure 1B, a 3D visualization of the SVF is provided. The Vector's length and color provide information about the strength of their weights. Shorter, darker vectors stand for weaker synaptic weights and longer, brighter vectors, indicate strong synaptic weights. For clarification, only 5% of the vectors are visualized in Figure 1B and the length of all vectors has been doubled to increase their visibility. However, this does not affect their expressiveness as their relative length is still meaningful with respect to their strength. It can be seen that the vectors are pointing in the direction of the start.

#### 2.1.3. Path Search

The synaptic weights, building up the vector field, are interpreted as forces used to move the agent and thus generate a path. The resulting path naturally leads away from obstacles as synapses connecting two neurons of the free space are stronger than synapses between free and occupied neurons. By averaging over the local vectors at each step local minimas can be avoided. The resulting force vector is subsequently added to the previous movement direction.

### 2.2. Tools and Techniques

**Figure 2:**
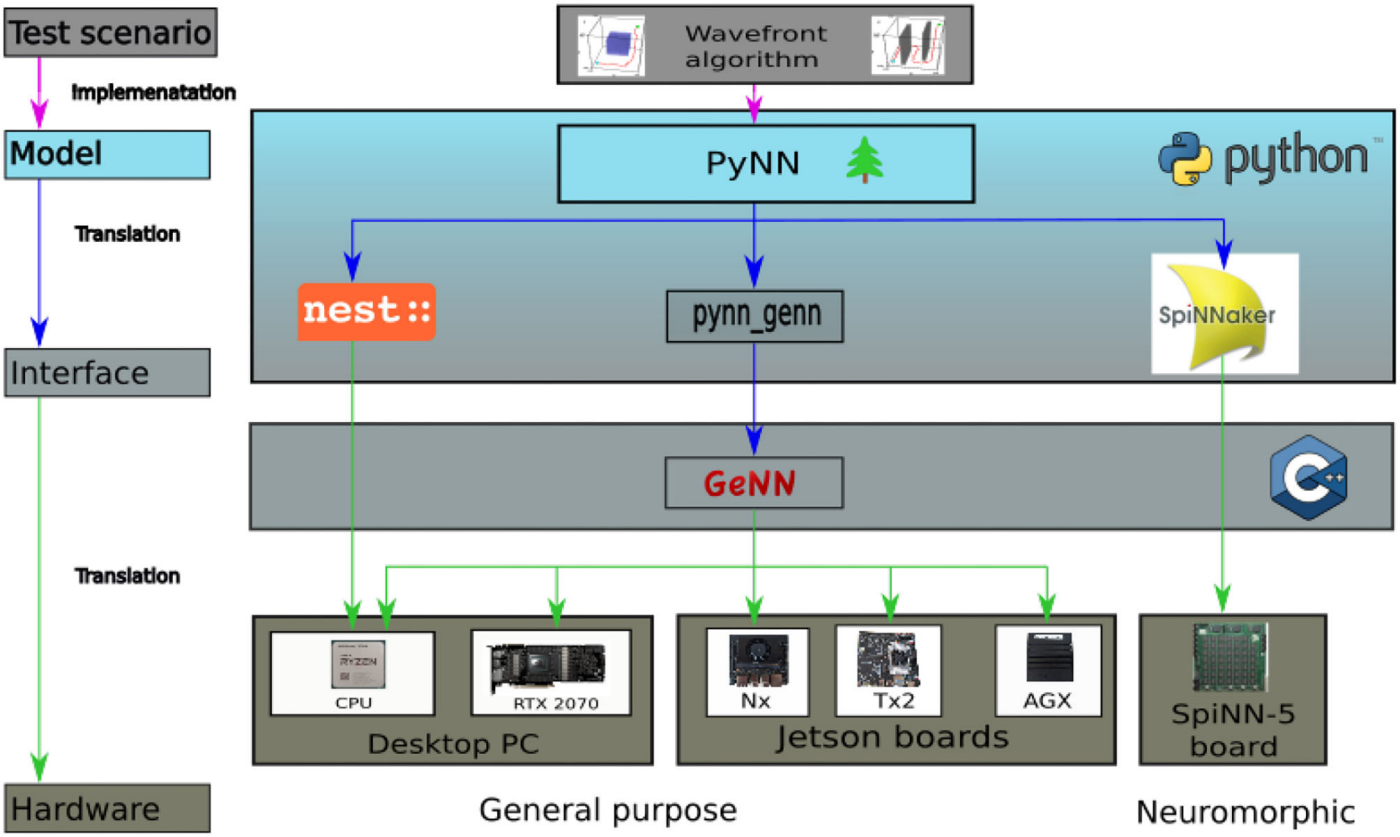
Overview of the hardware and software applied for this benchmark. On the top level the Wavefront algorithm representing the test scenario is featured. In the model layer below it is visualized how the algorithm is implemented via PyNN running in python. On the interface level the model is translated to the respective back ends and subsequently transferred onto the final hardware layer for execution.

Three different simulators, or rather hardware solutions, have been selected for the benchmark, representing three strongly deviating approaches for simulating spiking neurons. NEST is chosen as an actual simulator and the SpiNNaker system as a representative for neuromorphic architectures. GeNN constitutes a recently developed alternative running on conventional parallel hardware. GeNN can be used on a GPU but also in a CPU-only mode, which allows it to run on a broad range of hardware from desktop PCs to embedded systems. We evaluate GeNN with both, its CPU-only and GPU version. The CPU-only mode allows to compare the performance to the results obtained with the NEST simulator which were presented in Steffen et al. (2020). Both, GeNN on CPU and NEST, are run on a single processor core. A visualization of all applied hardware and software solutions is provided in Figure 2. As this paper aims to evaluate the performance of systems in context of robotics, the Jetson series by Nvidia is chosen as a hardware backend for GeNN on GPU. The Jetson series consists of several different embedded GPU systems, which were designed with the goal of supporting AI solutions in hardware with a small form factor. This enables the Jetson chipset to be integrated into mobile units (Franklin, 2018). The boards are general purpose hardware and are both more widely available and cheaper than the specialized neuromorphic hardware. Three different Jetson boards are evaluated in this benchmark, the Jetson Tx2, AGX Xavier, and Xavier Nx. In order to make the results of the embedded systems more comparable, a regular desktop PC is included in the analysis. The desktop PC is also used to run the NEST simulations. The PC has 32 GB of RAM and contains an Nvidia RTX2070 GPU and an AMD Ryzen 7 3700x CPU. A SpiNN-5 Board is used to run the SpiNNaker implementation.

#### 2.2.1. Hardware Specific Adaptations

In order to allow a fair comparison between the simulators, the benchmarking scenario needs to be implemented in a similar way on all platforms. PyNN offers the possibility to use the same model for all simulators. However, as noted in Diamond et al. (2016) this implies the risk that individual strengths of the simulators are not accounted for. Despite this issue, in this paper, all SNNs are modeled with PyNN, whereas Ostrau et al. (2020) opted to use the Cypress library and Diamond et al. (2016) decided to model their networks in the native modeling languages of the simulators instead of using their PyNN interface. However, using PyNN to model all networks allows to simulate exactly the same model on all backends. Figure 2 provides an overview of the hardware and software applied for this benchmark.

The benchmark is based on the NEST implementation of Steffen et al. (2020). Since all three models are implemented in PyNN, in theory it would be sufficient to specify a different PyNN backend. However, the different backends implement divergent subsets of PyNN's functions and models. Therefore, changes to the code are required to run the simulation on the different backends. However, it is also note-worthy that there are some possibilities to tailor the simulation more to the individual system. This allows to better account for individual strengths and weaknesses of each architecture. A big difference in the implementation of the simulators is the step size. While SpiNNaker uses a time step of 1 ms, GeNN and NEST simulations are usually run at time steps of 0.1 ms. This is due to the fact that 0.1 ms is the time step most often used for neuroscientific simulations (van Albada et al., 2018). The default time step is chosen for each simulator. The Wavefront algorithm is intended to solve a path planning problem in a time efficient manner, therefore, artificially slowing down the simulations in GeNN and NEST would not be realistic and distort the results. The time step of the simulation running on SpiNNaker is not lowered to match that of the other two simulators, as this would result in a slowdown of the simulation by a factor of 20. To account for the different time steps chosen, different total simulation times are required to allow the simulations to finish on the different platforms.

The original implementation of the neural wavefront algorithm presented in Steffen et al. (2020) uses the IF_cond_alpha model, which is a standard model in PyNN. It implements a LIF neuron with an alpha function to describe its post synaptic potential. This neuron model is currently not supported in sPyNNaker. The neuron model was thus changed to IF_cond_exp, an LIF neuron with an exponentially decaying post-synaptic potential. The SpiNNaker system has an additional constraint, the representation of synaptic weights as 16 bit integers. To convert the weights, a bit shift operation needs to be performed (van Albada et al., 2018). The algorithm used to determine the bit shift, does not allow the original maximum synaptic weight of *w*_*max*_ = 4000.0 μS. The highest maximum weight that can be implemented is *w*_*max*_ = 63.0 μS. The Wavefront Algorithm uses STDP with additive weight dependence. The value of *w*_*max*_ = 63.0 μS is too low, for the additive weight dependence to induce enough weight change to create the correct SVF. Therefore, for the implementation in sPyNNaker, the maximum weight is scaled down to *w*_*max*_ = 63.0 μS. After the simulation, the weight is scaled back up by a factor of *f*_*scale*_ = 4000.0/63.0. The re-scaling is not entirely correct, as the STDP rule introduces an additional term that cannot be properly re-scaled by this method. It would be possible to re-scale the weights correctly by sampling the weights before and after simulation, calculating Δ*w* and scaling it independently. However, this would introduce additional overhead in the pathfinding phase of the algorithm. In practice, the re-scaling, even though not entirely correct, still produces acceptable results. In the original implementation, the target neuron which starts the neural wavefront receives a DC current as an input. The current increases the neuron's potential inducing a spike. DC current sources are currently not implemented in sPyNNaker. Hence, the spike in the target neuron is induced using a neuron population of type SpikeSourceArray. The SpikeSourceArray population can then be connected to specific neurons via a projection. In this implementation the SpikeSourceArray population consists of one neuron that is connected to the target neuron with a static synapse. Instead of building up the membrane potential in the target neuron, a spike is induced right away. This is advantageous as the wavefront can be initiated right after the first time step. The spike source array introduces a small overhead for the simulation time for small maps on pynn_genn. To induce almost instantaneous spiking, the current in the DC source is set to 1,000 mV. A complete overview about all system specific adaptations is given in Table 1.

**Table 1:** Overview of all system specific adaptations for each NEST, SpiNNaker, and GeNN, in respect to the original implementation of Steffen et al. (2020).

**Features**	**Original implementation**	**NEST**	**SpiNNaker**	**GeNN**
Neuron model	IF_cond_alpha	IF_cond_exp	IF_cond_exp	IF_cond_exp
Step size	0.1 ms	0.1 ms	1 ms	0.1 ms
Weights	Unscaled	Unscaled	Scaled	Unscaled
Spike source	DC source	DC source	SpikeSourceArray	DC source

#### 2.2.2. Measurements

In order to gather data about performance, several measurements take place inside the simulation code. The individual functions Create neurons, Create synapses, Simulation, Build SVF, Compilation/Load Simulation, and Finding path are timed with the help of a python wrapper function. It saves the time stamp before the function is started and again when the function terminates. The delta is the execution time of the function and labeled with the function's name. In NEST the initialization of neurons and synapses in the code causes them to be initialized as soon as their respective definitions are executed. With sPyNNaker and pynn_genn the values are instantiated as well, however, when the run() function is executed, the synapses are instantiated again. This is due to the fact that they need to be placed onto the vertices of the MachineGraph in case of sPyNNaker, and executed in C++ or CUDA C in case of pynn_genn. The run() function combines the loading and running of the simulation in sPyNNaker and the compilation and running of the simulation for pynn_genn into one step. This makes it impossible to determine the exact time needed for the individual steps with the help of the wrapper. To determine the time required for loading and compilation, the sPyNNaker and GeNN simulation are initially run for 0 s. This triggers the loading and compilation respectively, but does not start the wavefront. When running the simulation on SpiNNaker again, the entire loading process is restarted. To avoid that the loading time is measured twice, the created logs of the SpiNNaker machine are read out. The logs contain timestamps that allow to determine correct start time. This timestamp is then used with the end timestamp of the python wrapper to determine the simulation time. The path length is determined during simulation time by obtaining the length of the list containing the path.

#### 2.2.3. Map Scaling

To compare how the different hardware solutions handle an increasing number of neurons and synapses, the networks are scaled up. As the network is a direct neural representation of the environment, enlarging the maps implies a likewise grow of the neural embodiment. The original maps, used in Steffen et al. (2020) of size 20 × 20 × 20, served as the reference value for the smallest maps. How an enlarged map influences the number of neurons and synapses within the network is visualized in Figure 1C. The number of synapses increases exponentially while the number of neurons is increased in a cubic manner, when the size of the map is scaled up. A prior examination regarding the maximum map size supported on each hardware specific implementation is required. The map size is limited by the available memory of the respective hardware architecture, for GeNN und NEST. For SpiNNaker, the long simulation times associated with larger maps limited the size of the network.

### 2.3. Metrics

To measure the performance of the applied hardware solutions, several metrics are introduced. As stated in Vineyard et al. (2019) it is practically meaningless to compare parallel architectures using the same metrics as applied to conventional VAs. Due to the architectural approaches being designed and optimized for different use cases, it is challenging—but absolutely necessary—to choose appropriate metrics enabling a solid understanding of their advantages and trade-offs.

#### 2.3.1. Simulation Time

It is not insightful to compare only internal metrics of the systems such as the speed of the processor clock. Instead, a comparison indicating how the systems perform when given an actual task is required. The Wavefront algorithm aims to solve the pathfinding problem as fast as possible and ultimately in real-time. Hence, the most interesting metric to consider is the execution time. When considering the time, not only the actual execution time is of interest, but also the amount of time needed to load or compile the simulation.

#### 2.3.2. Energy Consumption

The second meaningful metric is the energy consumption, more precisely, the average energy needed per run. For robots in general—but particularly for mobile robots—it is important that the individual components do not consume much energy. The measurement of the energy consumption is carried out externally. In literature, two methods for obtaining data regarding energy consumption are common. Firstly, a consumer grade power meter that allows to store time stamped data which can be extracted via an SD card. In Ostrau et al. (2020), the Ruideng UM25C power meter and PeakTech9035 power meter are used. Secondly, image recordings of the power meter with subsequential digital post-processing. In Diamond et al. (2016), van Albada et al. (2018), and Knight and Nowotny (2018) cameras are used to record the display of the power meter. A digital post-processing is required to obtain meaningful energy data.

For this benchmark, the energy consumption is measured using a household energy meter of type Voltcraft 4000 Energy logger that allows to store and extract time stamped data. The power meter has a minimal resolution of 0.1 W and an accuracy of ±1% and one count for the expected power draw. The power meter samples the power draw only once per minute and saves the data internally with a time stamp. The internal storage is extracted with the help of an SD card and then subsequently converted into csv format by the Voltsoft software that comes with the Voltcraft 4000 Energy Logger. In order to obtain the total energy consumption, the values are integrated using the numpy trapz function. The time step is set to 60s.

#### 2.3.3. Path Length

The path length is measured during simulation, after a path has been successfully determined. As the path is saved as a list, its longitude is simply the length of the list. To compare the length of the paths found on the different implementations, the median of the path lengths is taken over all runs on a particular map for each hardware solution. This allows to quickly determine if the path length differs throughout runs on the same simulator.

Comparing the path length determined on the different hardware solutions poses an issue. On SpiNNaker the weights differ because they are converted via bit shifting, causing some small errors, which are then amplified by the scaleup later in the process. Ultimately, we would use the path length to compare the accuracy of the different STDP implementations. In other words, we investigate if in case all simulators get the same initial weights, do they have the same final weights after learning via STDP.

#### 2.3.4. Hardware Resources

In addition to the main metrics, the consumption of different hardware resources, namely the memory usage and CPU/GPU of the Desktop PC and the Jetson boards are measured. This is done with the help of logging software. For the Desktop PC, glances^1^ is used for CPU and Memory as well as NVIDIA System Management Interface (nvidia-smi^2^) for the GPU data. On the jetson boards a logging script based on jetson-stats^3^ is run to log the memory usage.

## 3. Experiments and Results

### 3.1. Comparing Different Hardware Solutions

The total time of the simulation includes the time measurements of all functions that are needed to execute the Wavefront algorithm. Table 2 gives an overview of the median total time, path length, and average energy consumption per run. However, as the comprehensive version is very long and detailed, Table 2 is only an excerpt. It comprises SpiNNaker, NEST, and GeNN. The GeNN implementation was carried out on an AGX Xavier. The complete table including data for GeNN on RTX2070, in CPU-only mode, on a Tx2 and a Xavier Nx is provided as Supplementary Material. The detailed analysis in this paper is focused on map IV from Steffen et al. (2020), since this is the most complex. Tests on other maps presented in Steffen et al. (2020) show similar results, indicating that the statements can be generalized. As not to go beyond the scope of the work these additional results are not presented in this paper.

**Table 2:** An overview of the results of the simulations on the map IV.

	**Map size**	**Total time [s]**	**Path length**	**Average energy per run [J]**
GeNN	20	54.13	36.0	2254.65
	25	85.38	40.0	4358.44
	28	116.33	46.0	5521.26
	30	143.63	49.0	7169.49
	33	201.29	50.0	2347.63
SpiNNaker	20	64.62	32.0	13132.09
	25	128.50	49.0	23881.05
	28	171.26	44.0	14484.20
	30	162.46	50.0	13593.85
	33	259.03	53.0	21046.42
	35	324.96	55.0	24599.44
	40	368.47	72.0	30573.19
NEST	20	8.39	38.0	1861.54
	25	18.34	42.0	1908.04
	28	26.76	41.0	2453.65
	30	35.20	48.0	3089.95
	33	50.28	48.0	4294.51
	35	72.84	58.0	6126.01
	40	114.15	65.0	7641.54
	45	180.23	66.0	12266.63
	55	421.65	81.0	50946.67

*The median total time, path length, and average energy consumption per simulation run is listed for each implementations. The GeNN version was carried out on an AGX Xavier. Data regarding GeNN executed on other hardware solutions is given as Supplementary Material*.

The times for the individual functions Create neurons, Create synapses, Simulation, Build SVF, Compilation/Load Simulation, and Finding path are at first evaluated separately for every map. The median, which is more robust against outliers than the mean, and the standard deviation of the time are determined for every function separately. In order to obtain a total time, as provided in the second column of Table 2, the duration for the individual functions are summed for every run and subsequently.

The implementations running on the desktop PC are generally faster than implementations on other hardware. With total simulation times between 7.20 and 409.27 s, the implementation of GeNN on the CPU has the shortest total time of all implementations, followed by the implementation in NEST (8.39–421.65 s). Both implementations run on a single thread on the CPU of the desktop PC. The GeNN implementation running on the RTX2070 GPU has slightly longer total simulation times than the two implementations on the CPU. The implementations running GeNN on the Jetson Boards take significantly longer than the implementations on the desktop PC. The total simulation time of map size 30 on the RTX2070 is still considerably shorter than the total simulation time for map size 20 on any of the Jetson boards. The AGX Xavier has the best total simulation times out of the three boards, followed by the Xavier NX. The implementation running on the SpiNNaker board has worse total simulation times than the AGX Xavier, beating the Xavier NX and the Tx2, with the Tx2 being the slowest out of all hardware systems tested. The GeNN implementation running on the CPU scales better than the NEST implementation. Both are showing an exponential increase in total simulation time with increasing map sizes. For the AGX Xavier and Tx2 a similar trend can be observed, however, the trend is less noticeable due to the limited map sizes. For simulations with GeNN on the Xavier NX and the RTX2070, the total time appears to increase linearly. Some linear segments can be observed when looking at the scaling of total time on the SpiNNaker implementation. There is, however, a very striking deviation from the scaling, the total time decreases when the map size is increased from 28 to 30.

### 3.2. Performance Analysis of Isolated Functionalities

**Figure 3:**
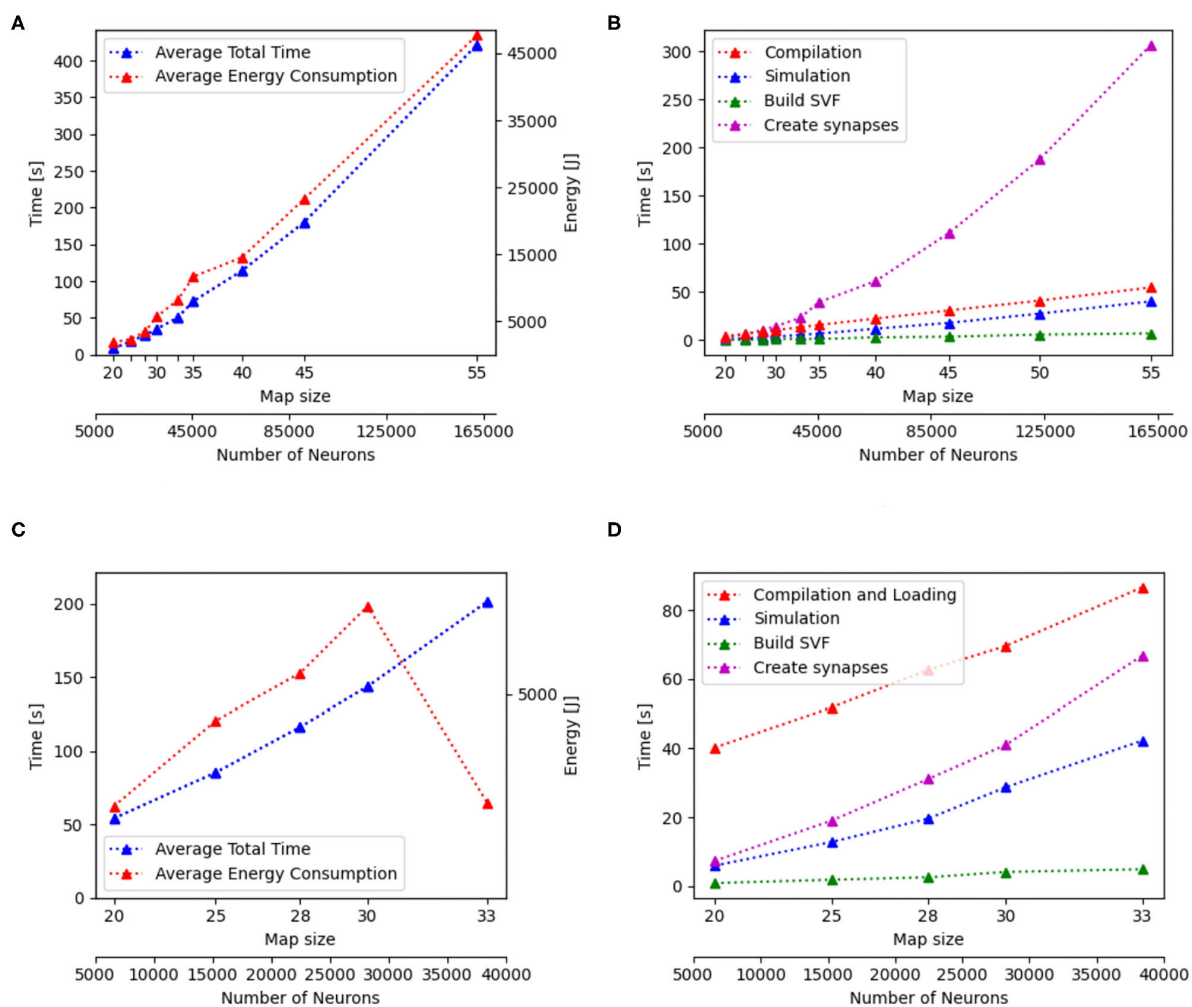
Scaling properties of functions for GeNN on the CPU in **(A,B)** and for Jetson AGX Xavier in **(C,D)**. **(A,B)** Show how the total time and energy consumption scale with increasing map sizes. In **(C,D)**, the development of the different functions of the Wavefront algorithm is displayed with regard to an increasing map size. The x-axes show the map size and thereby, the number of neurons.

**Figure 4:**
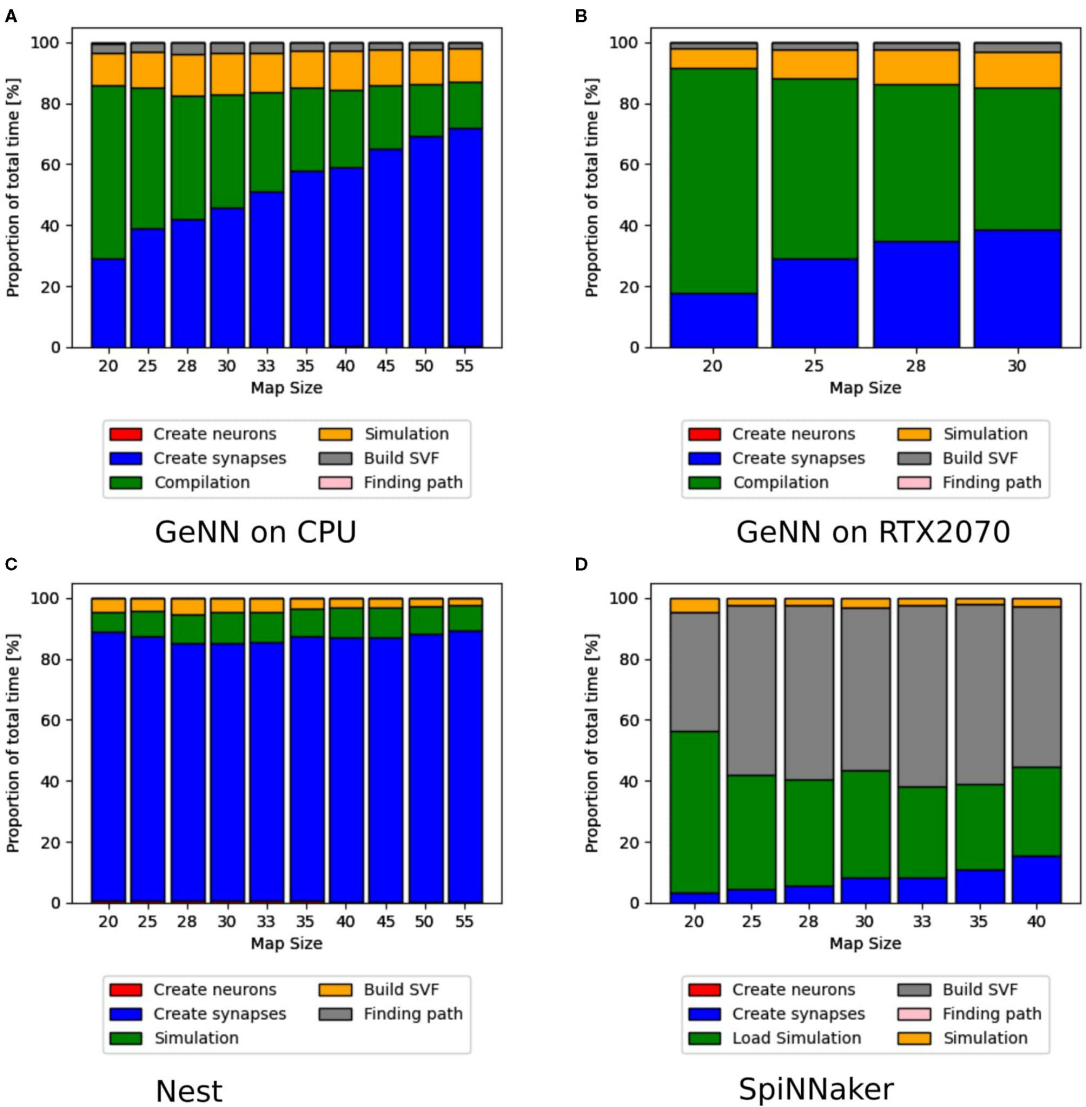
Distribution of total time simulated with GeNN on a desktop PC in **(A,B)** and NEST in **(C)** and SpiNNaker in **(D)**. The different proportions of the sub-functions in percent are shown as bars for every map size. For **(A,B)**, the compilation time, shown as a green bar, make up the highest proportion of the total time. With increasing map sizes, the proportion of synapse creation increases. In **(B)** (RTX2070), the proportion of the actual simulation also increases steadily, whereas it remains more constant in the CPU implementation in **(A)**. In **(C)**, the NEST implementation, the synapse creation has by far the highest proportion of the total time. For the SpiNNaker implementation in **(D)**, the functions to load the simulation and build the SVF form the highest proportion of the total time.

Additionally to the total time measurements in Table 2, Figure 3B shows how the individual functions scale with increasing map size for the implementation of GeNN running on the CPU. One can see that the exponential component in the scaling of total time is caused by the creation of synapses. In Figure 4A, the proportion for synapse creation is shown to increase considerably with increasing map sizes, eventually outweighing compilation time. For a map size of 55, synapse creation makes up 75.01% of the total time. On smaller maps, the compilation time dominates the total time. The proportion of the function Simulation increases only slightly with an increase in map size. Except for the creation of synapses, all other sub functions increase linearly with increasing map sizes. Compared to the implementation of GeNN on the RTX2070, the compilation times for GeNN on CPU are shorter. As shown in Figure 4B, on the RTX2070, compilation and loading times make up by far the largest fraction of the total time with 77.63% for a map size of 20. The proportion of compilation and loading time decreases with increasing map sizes, however, it still makes up almost 50% of the total time. Creating synapses takes more than twice as long as the simulation of the SNN itself for maps of all sizes. The development of the individual functions is illustrated in Supplementary Material. All sub functions scale linearly with increasing map sizes, except for the synapse creation, where the beginning of an exponential increase can be observed.

**Figure 5:**
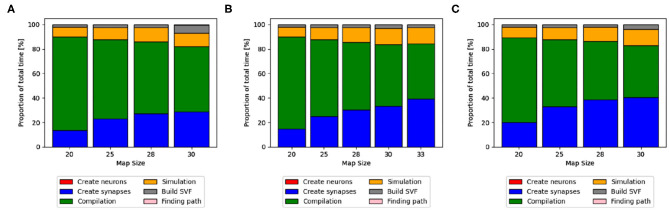
Distribution of total time on Jetson boards. The different proportions of the sub-functions in percent are shown as bars for every map size. Compilation and loading time, shown as a green bar, make up the highest proportion of the total time. With increasing map sizes, the proportion of synapse creation (blue bar) and simulation (orange) increases. On the Xavier Nx a sharp increase in the proportion of the time to build the SVF (gray) can be observed for map size 30. **(A)** Xavier Nx, **(B)** AGX Xavier, **(C)** Tx2.

**Figure 6:**
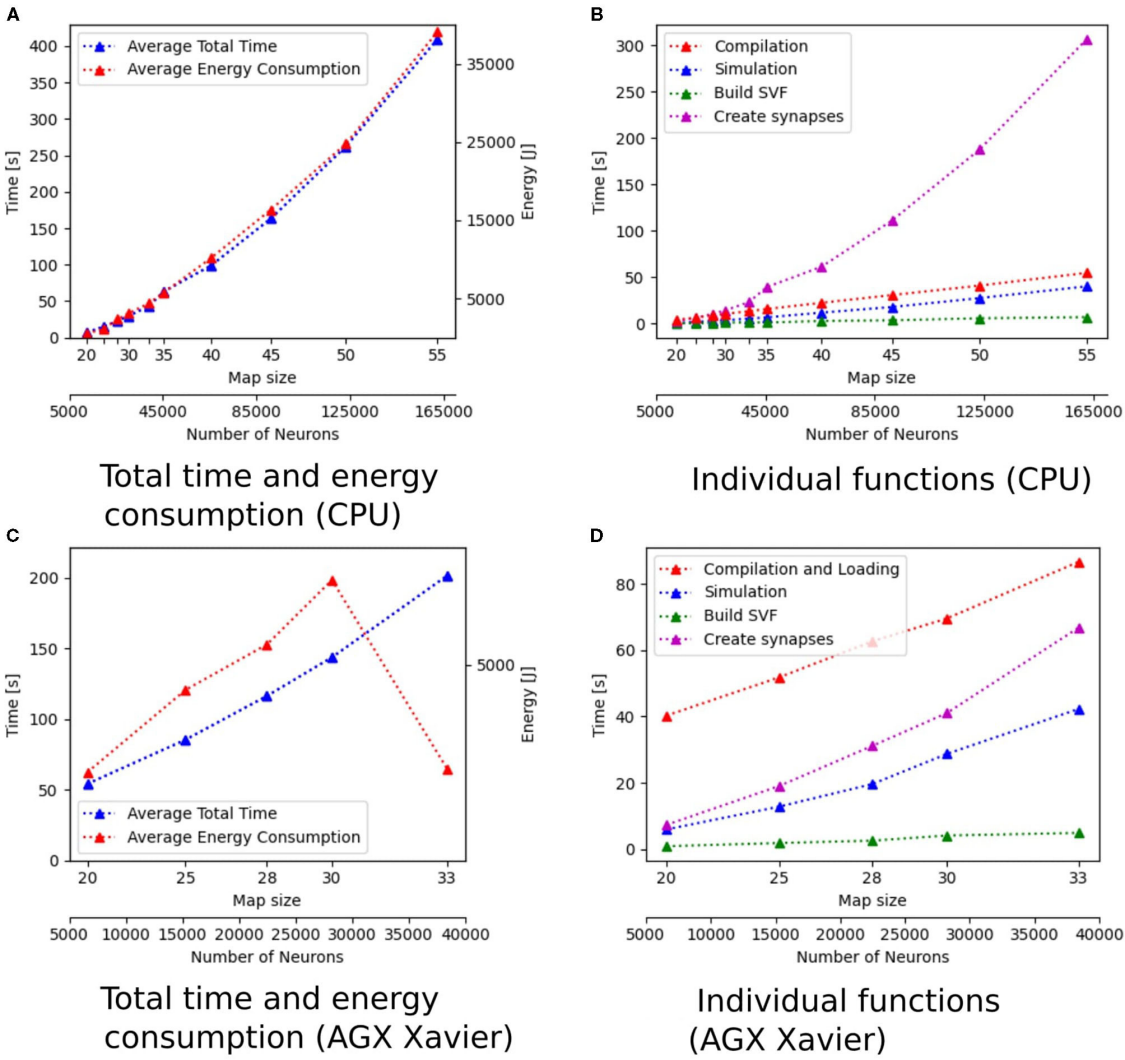
Scaling properties of functions for SpiNNaker in **(A,B)** and NEST in **(C,D)**. **(A,C)** Show how the total time and energy consumption scale with increasing map sizes. In **(B,D)**, the development of the different functions of the Wavefront algorithm is shown with regard to an increasing map size. The x-axes show the map size and the number of neurons.

The proportions of individual functions on the Jetson boards show a pattern similar to the RTX2070. Figures 3B,D, **6B,D** show how the time of individual functions scale with respect to increasing map sizes^4^. The function Compilation and loading and Simulation show a linear increase for the Tx2 and the AGX Xavier, whereas Create synapses shows the beginning of an exponential increase when the map is scaled up. On the Jetson Xavier Nx, the function Create synapses scales linearly. The time required for compilation/loading reaches a plateau at maps of size 30. The time required to build the SVF remains almost constant on the AGX Xavier for all map sizes. For the Tx2 and Xavier NX, however, a strong increase is measured for the largest map size. The proportions of the different sub-functions are visualized in Figure 5. With 76.6, 73.8, and 69.3% compilation and loading time represents the largest part of the total time on all three Jetson boards for map size 20. The proportion of the compilation and loading time decreases when maps get larger, however, they still make up around 45% of the total time and are almost two times higher than the actual simulation of the SNN. The function to create synapses also takes longer than the simulation time itself. With exception to the Xavier Nx on map size 20, synapse creation takes more than twice as long as the actual simulation for all Jetson boards on all map sizes. The time required to build the SVF increases with larger maps in proportion to the total time. On the Xavier Nx, in particular, a strong increase can be observed for maps of size 30.

The latest update of pynn_genn introduced the reuse_model flag. I allows for the CUDA backend to reuse the model of a previous run of a simulation, if the same network is used. Most of the generated code can therefore be reused and does not need to be compiled again, thus significantly saving compilation time. For the simulation running on the RTX 2070 the compilation and loading time could be reduced by 8.01 s for the smallest map and 7.84 s for the largest map which accounts for 72.1 and 44.4% of the simulation and loading time. For the Jetson AGX Xavier a reduction by 24.51 s for the smallest map and 27.76 s for the largest map were observed which amounts to a reduction by 61.9 and 22.1% of the simulation and loading time.

Figure 4C shows that for the NEST implementation. The total time is dominated by the creation of synapses which makes up between 84.3 and 89% of the total simulation time for all map sizes. This reflects the results in Steffen et al. (2020), where the creation of synapses also contributes the most to simulation time. For the SpiNNaker implementation (see Figure 4D), the simulation and loading times far outweigh the time it takes to create the synapses, which is very similar to the observed behavior of GeNN on the CPU (Figure 4A). Similar times for Create synapses are expected, as the function is executed on the CPU of the desktop PC in both cases. However, on the SpiNNaker board, Create synapses makes up a much lower proportion of the total time, ranging from 3.3% to 15.6% for the smallest and largest map, respectively. The time it takes to load the simulation and to build the SVF makes up the highest proportions of the total time. As illustrated in Figure 6B, the sudden decrease of total simulation time, that can be observed in Figure 6A, appears to be mainly due to a decrease in the time it takes to build the SVF. It contributes up to 59% to the total time, which is a much larger proportion than the NEST implementation or the GeNN implementations running on the desktop PC.

On all systems, the time it takes to create neurons is negligible compared to all other functions which is not surprising as the number of neurons is only a fraction of the number of synapses and weights. The function Path finding also makes up only a small proportion of the total time.

### 3.3. Path Length

**Figure 7:**
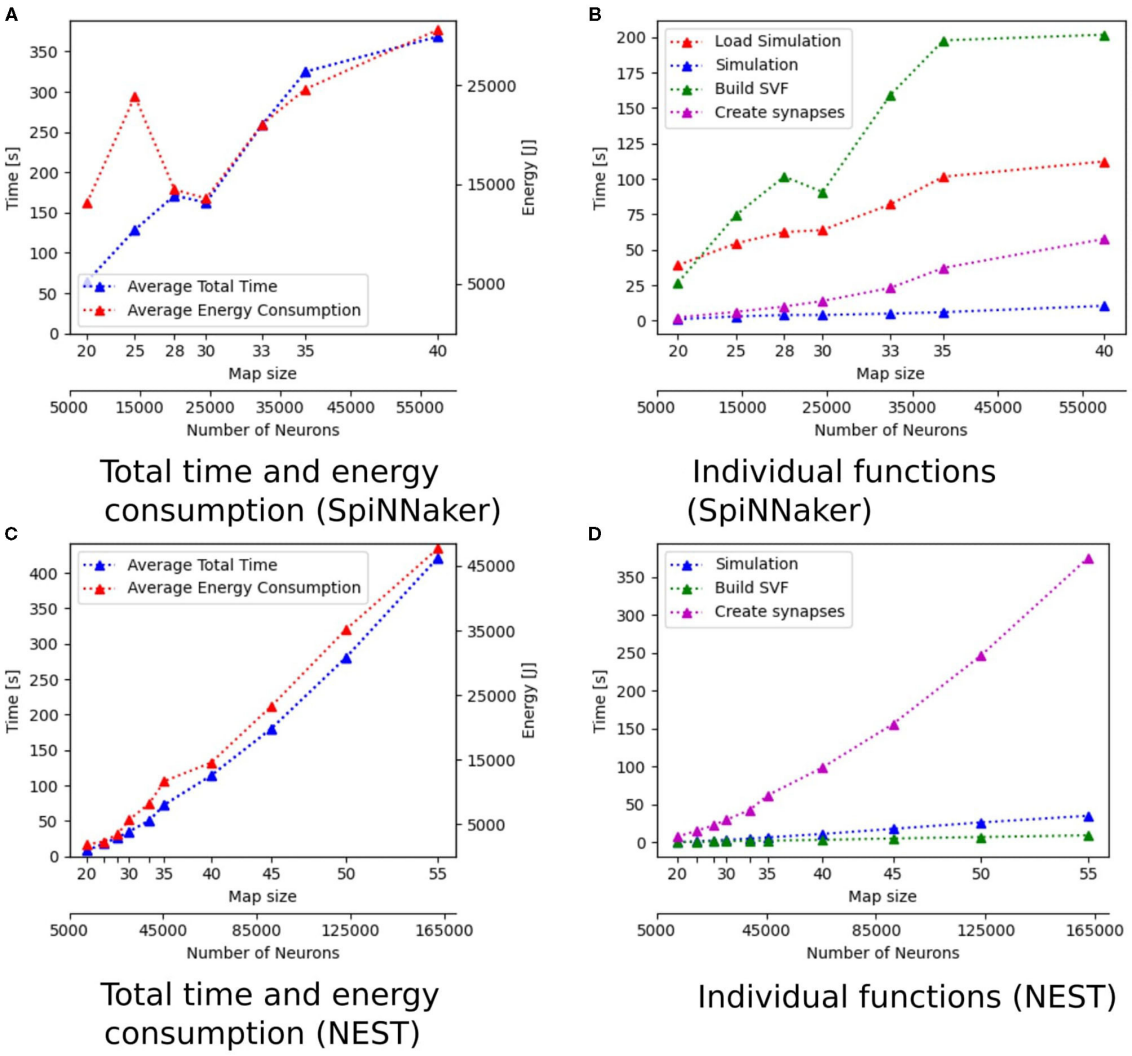
Path found for size 20 and 25 with GeNN on CPU. The start neuron is highlighted in green, the target neuron cyan, and the path orange. For map size 25 a more direct path is found, which causes the path length to be slightly shorter, despite the larger map size. **(A)** Path for map size 20. **(B)** Path for map size 25.

The path length, stated in the third column of Table 2, is measured during the simulation, after the pathfinding process is finished. The path is saved as a list, which means the path's length equals the list's length. To compare the length of the paths found on the different implementations, the median of the path lengths is taken over all runs on a particular map. To check if the path length differs between individual runs, the standard deviation of the path lengths is considered as well. Since the Wavefront algorithm does not guarantee to find an optimal path (Steffen et al., 2020), the path for map size 20 has more detours than the path in the larger map. This is visualized for GeNN on PC in Figure 7.

The wavefront Algorithm relies on the weights of the synapses to create the SVF and find a path. In order to make the path length comparable, it needs to be ensured that the simulators start out with the same initial weight. Unfortunately this can only be ensured for the simulations in NEST and GeNN, as the bit shifting in the SpiNNaker system causes rounding errors, which are amplified by the scaling of the weights. As expected the path lengths differ between the SpiNNaker implementation and the other implementations. However, there is also a difference between the NEST and GeNN implementations. The most striking observation is that the path lengths differ on different GeNN implementations. Path lengths on the Jetson boards even differ between individual runs on the same map and same Jetson board. To rule out that the different GeNN implementations start out with different parameters, the initial synaptic weights are compared. The initial weights are measured after the code is compiled and loaded onto the GPUs. This ensures that values are not altered during compilation or initialization on the GPU. All GeNN implementations share the same initial weights. To further narrow down the cause of the different path lengths, the final weights are analyzed as well. As expected, the final weights differ between the Jetson boards and the GeNN implementations on the PC. The final weights also differ between runs on the maps on all three Jetson boards. The differences in the path length, hence arise during the simulation of the Wavefront algorithm.

### 3.4. Energy Measurement

The data about power consumption, as provided in the fourth column of Table 2, is stored with a timestamp associated with every data point. To get the relevant data for each map, the power measurement data is matched with the timing data, by comparing their timestamps. All power draw data with timestamps between timestamp_start of the first run and timestamp_end of the last run are considered. As the power meter only logs power draw once a minute, very few data points are available for every map. The data is linearly interpolated and integrated to obtain the total energy. The total energy obtained is then divided by the number of simulation runs to get an average value for the energy consumption of a single run.

The energy consumption depends on the total time and the energy efficiency of the applied hardware. Figures 3A,C, 6A,C show how the total time and energy consumption per run increase with an up scaled map size for GeNN on the CPU, GeNN on an AGX Xavier, the SpiNN-5 board and in NEST. It can be observed that the average energy consumption is increasing very similarly to the total time. The AGX Xavier and Xavier NX both require less energy per run than the GeNN implementations on the desktop PC. This is despite the fact that both Jetson boards have a much longer total time. The GeNN implementation on CPU consumes less energy than the implementation on the RTX 2070 which correlates with the shorter total simulation times.

### 3.5. Hardware Resources

**Figure 8:**
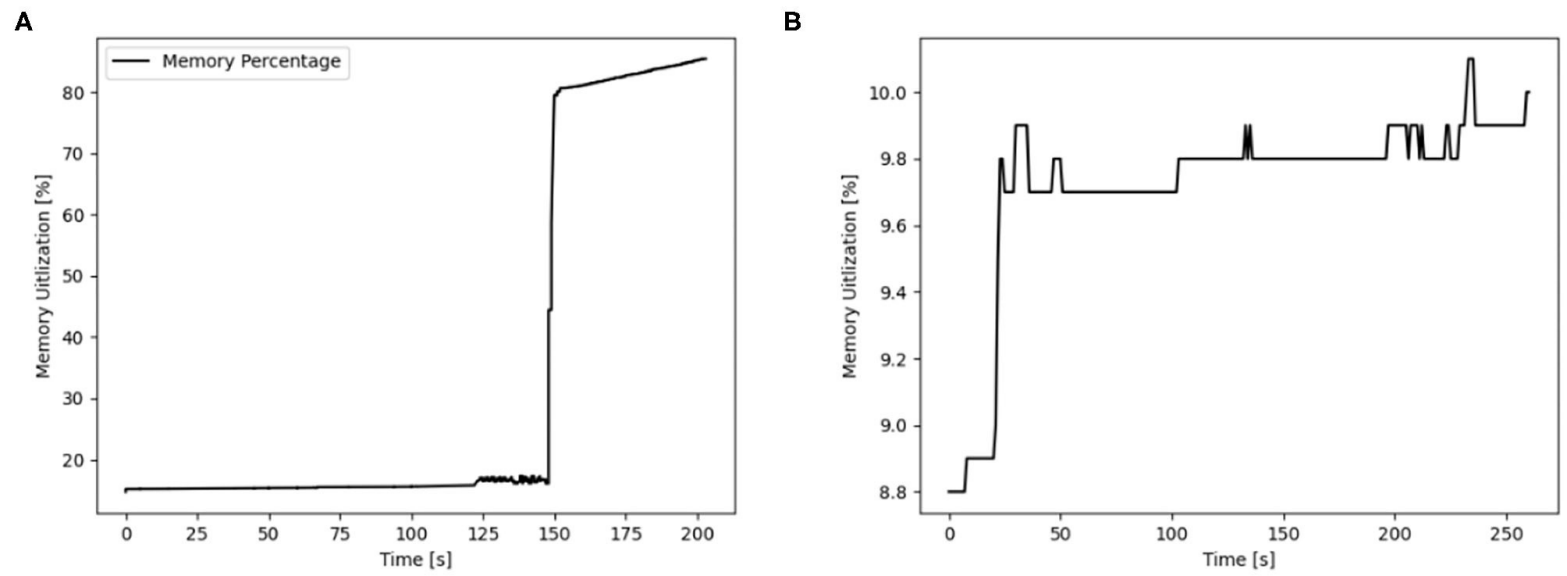
Memory utilization in percent for GENN (exemplary AGX Xavier) and neuromorphic hardware for the map size 33. **(A)** AGX Xavier. **(B)** SpiNNaker.

**Figure 9:**
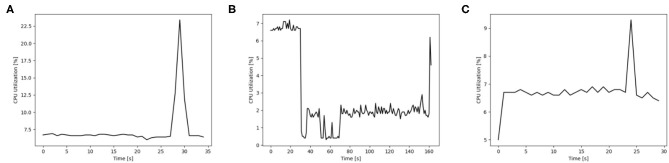
CPU utilization in percent for different simulation options of GeNN on a Desktop PC and additionally on neuromorphic hardware for the map size 30. **(A)** GENN (GPU), **(B)** SpiNNaker, **(C)** GENN (CPU).

The usage of hardware resources shows similar development for the different simulators on the different map sizes, therefore resource use is discussed by using exemplary data. Additional data is provided as Supplementary Material. The development of memory usage is similar for all GeNN simulations. Figure 8 shows memory utilization in percent for the Jetson AGX Xavier which is similar to the other GeNN simulations and SpiNNaker for map size 33. For the Jetson AGX Xavier there is a very slow steady increase in memory usage, until the simulation is compiled and loaded which causes a sharp rise in memory utilization and is followed by a slower steady increase during the simulation itself. For SpiNNaker the memory usage rises much earlier, during the creation of the synapses and then rises again sharply when the simulation is loaded. The development of the CPU usage on the Desktop PC differs between the different simulators. Figure 9, shows the CPU utilization in percent for GeNN on CPU, GeNN on the RTX2070 and for SpiNNaker. For GeNN on the RTX270 a large single spike can be observed that takes place during compilation and loading of the simulation. For the SingleThreaded CPU backend of GeNN a large spike and a second smaller spike can be observed which coincide with compilation ad loading of the simulation and the simulation itself, respectively. Curiously, a larger proportion of the CPU is used during the compilation and loading process of the CUDA backend. For SpiNNaker one can observe a large spike in CPU usage during the creation of synapses and during the function Build SVF.

## 4. Discussion

The fact that there is no significant difference in path lengths between the different implementations shows that the mechanisms to simulate STDP produce similar and consistent results. The Jetson boards form an exception in this regard as they have different path lengths in different runs of the simulation with the same map size. This difference is most likely caused by the non-associative nature of floating point numbers, which can lead to different results when additions are parallelized in GPUs. This makes it difficult to compare results obtained from calculations on a GPU to results obtained from calculations on CPUs (Whitehead, 2011). The possibility of simulation results differing between runs of a simulation, especially when STDP is used, is described in Yavuz et al. (2016). In Knight and Nowotny (2018), no noticeable divergence between simulations of the microcircuit mode were reported. In our benchmark only a single spike wave is simulated, making the model more susceptible to small differences in the simulation. However, it is surprising that the result deviations only appear on the embedded systems and not on the discrete GPU. Particularly as both are used with the single precision floating point operations, which makes the deviations in the simulation results more likely than double precision operations.

The Jetson boards, representing edge computing, stand out with regard to energy efficiency. As they are designed with a use for mobile applications in mind, they are optimized for low power draw. A desktop PC on the other hand is not optimized in this regard. This explains the high energy consumption per run on the implementations on the desktop PC, despite the much shorter total simulation times. The NEST and GeNN implementation running only on the CPU of the desktop PC need less power than the GeNN implementation on the RTX2070 as their simulation times are shorter and the GPU is an additional device requiring energy. The high energy consumption of the SpiNNaker implementation shows that hardware with a higher power draw, that at the same time is able to run the simulation faster can still be more efficient than systems that have a low power draw but longer simulation times.

### 4.1. Contextual Analysis

For all implementations of GeNN, compiling/loading of the simulation and the creation of synapses are both tasks which are performed by the CPU. Therefore, for the majority of the time, only the CPU performs operations. Only the actual simulation of the SNN is carried out by the GPU. As the Jetson boards are designed for GPU heavy tasks, their CPUs are rather lightweight. The CPU complexes of all Jetson boards is made up of ARM based CPUs. For the Xavier Nx the CPUs can reach a maximum frequency of 1.9 GHz. A maximum frequency of 2.26 GHz can be reached by the CPUs of the AGX Xavier, while the CPUs of the Tx2 reach a maximum frequency of 2.0 GHz. These maximum frequencies are low, however, consumer CPUs are generally more powerful than embedded processors. This explains the large difference in compilation times between the implementations on the Jetson boards and the simulations of GeNN on PC. The difference in compilation time between pure CPU implementation and the one using the GPU can be explained by the fact that no transfer between host RAM and GPU memory needs to take place when only a CPU is used. Also, no additional CUDA code needs to be generated and compiled in the CPU only version of GeNN.

The implementation in NEST does not need to be compiled or loaded to an external device. However, it takes longer to create neurons and synapses than on the GeNN CPU implementation. As shown in Figure 1C, the amount of synapses drastically increases with increasing map sizes which also leads to an increase in the time needed to create them. The creation of synapses is performed on the CPU using python for all implementations. For GeNN implementations, this entails the instantiation of PyNN projections. However, the synapses need to be instantiated again later when the C++/CUDA code is run. The compilation and simulation portions of GeNN do not display any exponential increase when maps are scaled. This suggests that GeNN, when used with its native frontend, can cope significantly better with large numbers of synapses than when it is used with its pynn_genn frontend. This comes as no surprise as C++ is a compiler language while python is an interpreter language which tends to be slower than compiler languages. When comparing the time required to create neurons and synapses in the GeNN and NEST implementations one also needs to account the compilation time in GeNN, but even when doing so, neurons and synapses are instantiated faster on the GeNN implementation. This advantage becomes especially apparent for large map sizes where it takes more than 20 s longer for the NEST implementation to create the synapses then for the GeNN CPU implementation. This difference in the time it takes to construct the network causes the overall shorter total time of the GeNN implementation on CPU, as NEST has shorter times for all map sizes when comparing only the simulation of the network.

For the implementation on the SpiNN-5 board, a similar amount of time is spent on synapse creation as in the GeNN CPU implementation. However, as the functions Simulation, Load Simulation, and Build SVF take much longer, it is less noticeable. As described in Rowley et al. (2020), both simulation and loading require communication with the host system. This additional overhead explains the large difference in time required for simulation between the SpiNNaker system and the GeNN implementations. Especially the time it takes to build the SVF for the SpiNNaker stands out, as the SVF is built by the CPU of the desktop PC just like the implementations of GeNN on the desktop PC and NEST, which are all considerably faster. This can be explained by the fact that to build the SVF, the weights of all inhibitory- and excitatory connections are required, which need to be extracted from the simulation. For the implementation of GeNN on CPU and NEST, the weights are already present on the system RAM, hence requiring short loading times. These loading times get a bit bigger for GeNN on the RTX2070, where they need to be loaded from the device memory of the GPU. In the SpiNNaker implementation, the weights need to be transferred between the SpiNNaker device memory and the system RAM via a 100 Mbit Ethernet cable which is about 10 times slower than on the internal data busses used in the GeNN and NEST implementations.

### 4.2. Limitations and Outlook

The hardware performance comparison between high-performance GPU (RTX2070) and embedded GPU (Nvidia Jetson) can be seen as inappropriate. As one can foresee that the implementation running for desktop GPU are much faster while consuming more energy. However, it is still very interesting to see the exact delta of embedded GPU and discrete GPU/CPU. It provides a good insight about how far the state of development with embedded systems really is.

Memory is a limiting factor in this benchmark, as it limits the size of the SNN that can be simulated. Especially for the GeNN implementations that use the CUDA backend this poses a problem. Memory on GPUs tends to be less than the RAM on a PC. Regarding the Jetson boards, memory is shared between CPU and GPU which further limits their capabilities to simulate large SNNs, as there is an increasing performance penalty once the memory reaches its limits. The scripts that log the hardware data only allow for a sampling rate of 1 Hz as logging data more often than once per second results in uneven sampling intervals. Due to the low sampling rates of the energy logger, an in-depth analysis of the energy consumption and power draw is not possible. The data, however still shows trends and gives an order of magnitude of the energy consumption of the different hardware platforms. The implementation of models in pynn_genn introduces a large overhead on the GeNN implementations, as the synapses and neurons first need to be instantiated as pynn projections and populations before they can be simulated in GeNN which is implemented in C++.

As neuromorphic platforms with static synapses, such as True North or NeuroGrid, do not support neural plasticity, this benchmark using STDP is not well applicable for them. Platforms with configurable plasticity are generally capable of on-line learning. However, ROLLS does not support the required learning rule and the ODIN and TITAN chip have a quite limited number of neurons, only allowing the investigation of tiny maps. Platforms with programmable plasticity like BrainScaleS 1 & 2, SpiNNaker-2, and Loihi not only support online learning but also the required learning rule STDP. Therefore, our benchmark implementation is easily transferable to them. Hence, we want to transfer the implementation to either Loihi, SpiNNaker-2, or BrainScaleS 2.

## Data Availability Statement

The raw data supporting the conclusions of this article will be made available by the authors, without undue reservation.

## Author Contributions

LS, AR, and RD are responsible for the idea, the core concept, and the architecture of this paper. LS, RK, SU, and SN did the research and wrote the paper. All authors contributed to the article and approved the submitted version.

## Conflict of Interest

The authors declare that the research was conducted in the absence of any commercial or financial relationships that could be construed as a potential conflict of interest.

## Footnotes

^1^https://nicolargo.github.io/glances/.

^2^https://developer.nvidia.com/nvidia-system-management-interface.

^3^https://github.com/topics/jetson-stats.

^4^Plots regarding the scaling properties of GeNN on Tx2, Xavier NX, and RTX2070 (desktop GPU) are provided in Supplementary Material.
