# Supplementary material for: Benchmarking Highly Parallel Hardware for Spiking Neural Networks in Robotics
Source: Front Neurosci. 2021 Jun 29;15:667011. doi: 10.3389/fnins.2021.667011 (PMC8275645; doi:10.3389/fnins.2021.667011)
Supplement: Supplementary file 1 [file Data_Sheet_1.pdf]

Supplementary Material

1 SUPPLEMENTARY FIGURES

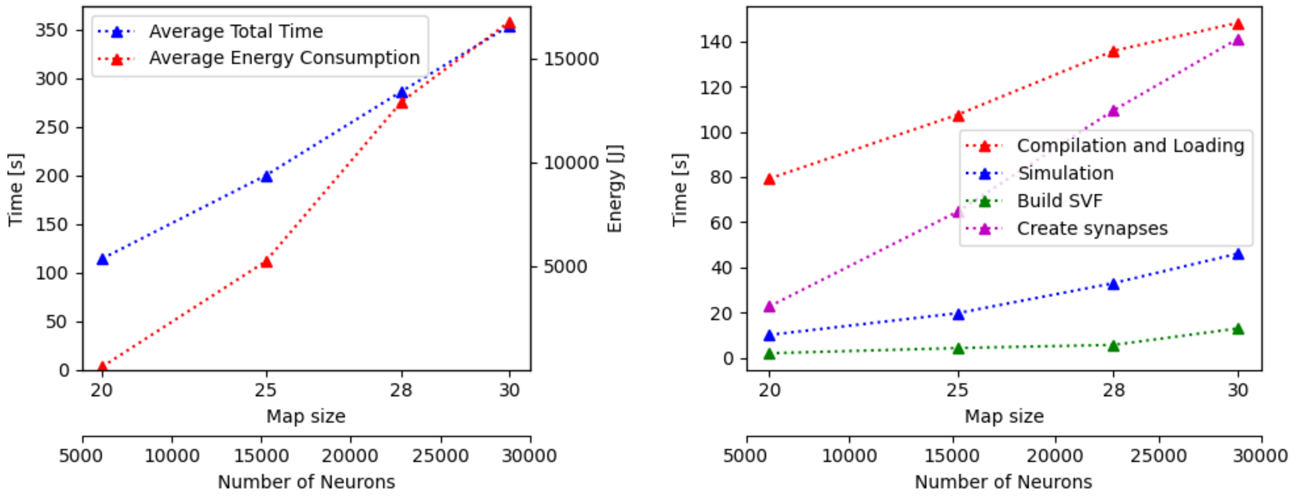

Figure S1. Scaling properties of functions for Jetson Tx2 on map IV.The left graphic shows how the total time and energy consumption scale with increasing map sizes. In the right graphic the development of the different functions of the Wavefront algorithm is shown with regard to increasing map sizes. The two x-axes of both sub figures show the map sizes and the number of neurons.

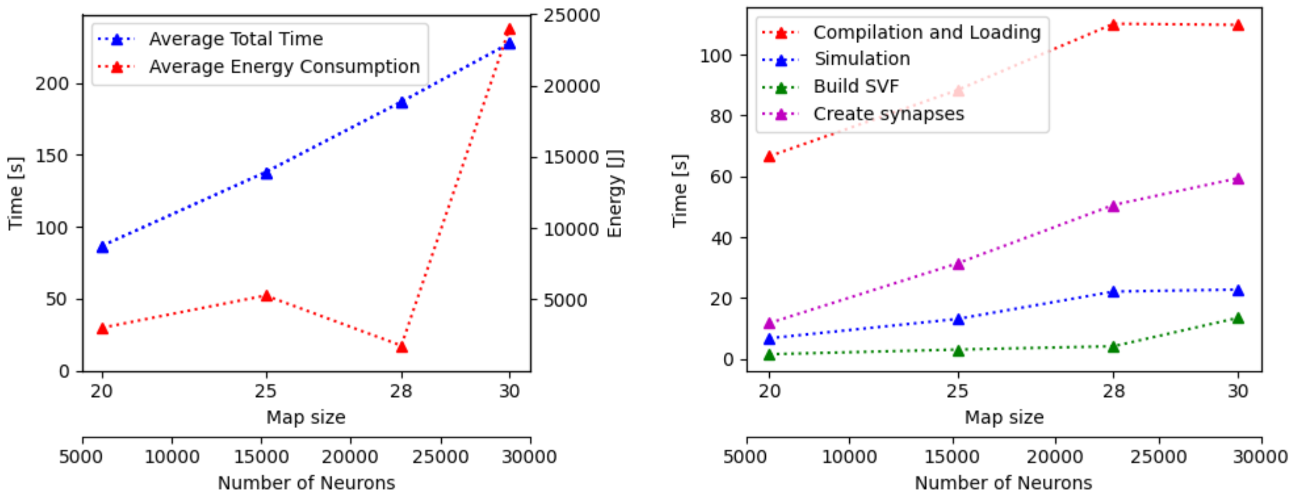

Figure S2. Scaling properties of functions for Jetson Xavier Nx on map IV.The left graphic shows how the total time and energy consumption scale with increasing map sizes. In the right graphic the development of the different functions of the Wavefront algorithm is shown with regard to increasing map sizes. The two x-axes of both sub figures show the map sizes and the number of neurons.

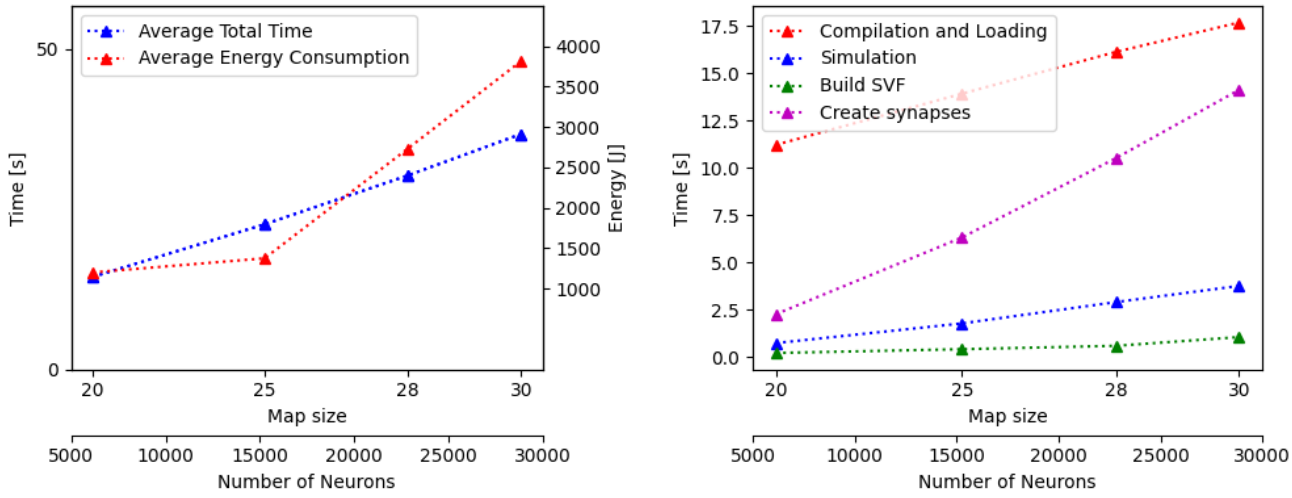

Figure S3. Scaling properties of functions for GeNN on the RTX2070 on map IV. The left graphic shows how the total time and energy consumption scale with increasing map sizes. In the right graphic the development of the different functions of the Wavefront algorithm is shown with regard to increasing map sizes. The two x-axes of both sub figures show the map sizes and the number of neurons.

2 SUPPLEMENTARY TABLE

	Map Size	Total Time [s]	Path Length	Average Energy per run [J]
GeNN on RTX2070	20	14.46	41.0	1201.82
	25	22.67	38.0	1376.56
	28	30.26	47.0	2728.85
	30	36.76	46.0	3817.19
GeNN on CPU	20	7.20	41.0	526.98
	25	15.15	35.0	1107.74
	28	23.11	47.0	2347.31
	30	29.77	49.0	3125.71
	33	43.29	46.0	4370.87
	35	63.59	55.0	5777.70
	40	98.67	58.0	10155.23
	45	163.36	60.0	16355.57
	50	262.22	69.0	24766.49
	55	409.27	79.0	39065.35
Jetson Tx2	20	114.22	41.0	143.54
	25	199.65	35.0	5213.75
	28	285.93	47.0	12894.51
	30	353.64	49.0	16762.27
Jetson Xavier Nx	20	86.86	34.0	3018.58
	25	137.98	37.0	5280.65
	28	186.83	46.0	1762.93
	30	227.51	48.0	24054.32
Jetson AGX Xavier	20	54.13	36.0	2254.65
	25	85.38	40.0	4358.44
	28	116.33	46.0	5521.26
	30	143.63	49.0	7169.49
	33	201.29	50.0	2347.63
SpiNNaker	20	64.62	32.0	13132.09
	25	128.50	49.0	23881.05
	28	171.26	44.0	14484.20
	30	162.46	50.0	13593.85
	33	259.03	53.0	21046.42
	35	324.96	55.0	24599.44
	40	368.47	72.0	30573.19
NEST	20	8.39	38.0	1861.54
	25	18.34	42.0	1908.04
	28	26.76	41.0	2453.65
	30	35.20	48.0	3089.95
	33	50.28	48.0	4294.51
	35	72.84	58.0	6126.01
	40	114.15	65.0	7641.54
	45	180.23	66.0	12266.63
	55	421.65	81.0	50946.67

Table S1. An overview of the results of the simulations on the map IV is shown. The median total time, path length and average energy consumption per simulation run is listed for all implementations.
